# Acupuncture attenuates the development of diabetic peripheral neuralgia by regulating P2X4 expression and inflammation in rat spinal microglia

**DOI:** 10.1186/s12576-020-00769-8

**Published:** 2020-09-23

**Authors:** He-yong Tang, Fan-jing Wang, Jun-long Ma, Hao Wang, Guo-ming Shen, Ai-juan Jiang

**Affiliations:** 1 Graduate School of Anhui, Anhui University of Chinese Medicine, No.1, Qianjiang Road, Hefei, 230012 Anhui China; 2 School of Integrated Traditional Chinese and Western Medicine, Anhui University of Chinese Medicine, No.1, Qianjiang Road, Hefei, 230012 Anhui China; 3 Anhui Province Key Laboratory of Research & Development of Chinese Medicine, Hefei, 230012 China

**Keywords:** Acupuncture, Diabetic peripheral neuralgia, P2X4, Diabetic rat

## Abstract

Diabetic peripheral neuropathy (DPN) is a chronic microvascular complication of diabetes. The purpose of this study is to find the underlying mechanism for the effects of acupuncture in DPN rats. Rats were rendered diabetic with a single injection of 35 mg/kg streptozotocin (STZ). These STZ-diabetic rats were treated with acupuncture for 20 min once daily. The therapeutic efficacy of acupuncture was assessed using mechanical withdrawal threshold (MWT) and thermal withdrawal latency (TWL) evaluations. After 14 days treatment, acupuncture markedly reduced the pathological injury in STZ-diabetic rats. Moreover, it significantly down-regulated P2X4 and OX42 expression along with the reduced levels of inflammatory factors (CXCR3, TNF-α, IL-1β, IL-6), GSP and lipid metabolisms in the spinal cord of the DPN rats. Acupuncture could relieve DPN in rats by regulating P2X4 expression and inflammation in spinal microglia.

## Background

According to traditional Chinese medicine theory (TCMT), diabetes mellitus (DM) is referred to as Xiaokezheng [1]. It belongs to the categories of “arthromyodynia”, “numbness”, “unfeeling”, and “blood arthralgia”, described by excessive drinking, eating, and production of urine [2]. The main type of DM is type 2 diabetes mellitus (T2DM), which patients often develop diabetic peripheral neuropathy (DPN) [3]. DPN is defined as “the presence of symptoms and/or signs of peripheral nerve dysfunction in people with diabetes after the exclusion of other causes” [4]. It affects sensory, autonomic, and motor nerve functions [5]. The symptoms of DPN include nocturnal burning or shooting pains in the legs and feet, indicating impairment or damage to small nerve fibers [6].

In the recent years, incidence of DPN among all chronic complications of diabetes has been reported to be relatively high [7]. Pharmacological therapy is effective in improving the clinical symptoms of DPN, but it is also associated with considerable side effects in a long-term drug treatment [8]. Compared with western medicine, specifically with applied pharmacological therapies, TCM provides effective treatment options with relatively mild adverse effects [9, 10]. Therefore, the use of TCM in the treatment of DPN is endearing a lot of attention among scholars, especially, the acupuncture [9]. As we know, acupuncture is an ancient Chinese method that used to treat diseases and relieve pain [11]. Acupuncture is a complex intervention that various disorders can effectively be cured by inserting long, fine needles into specific ‘‘acupuncture points’’ on the skin of the patient’s body [12]. There is some evidence that it may be beneficial in the management of DPN [6, 13–15]. However, the underlying mechanism of acupuncture action on DPN remains unclear.

Microglia are resident macrophages in the central nervous system, and they will immediately change from a "resting" state to an "activated" state after nerve damage [16]. Spinal cord microglia is also very important for the development and maintenance of DPN [17]. Tsuda and Inoue (2007) found that P2X4 receptors exist on microglia [18]. Previous studies showed that the P2X4 receptor (P2X4R), an ATP-gated purinergic receptor expressed on microglia, caused tactile allodynia after nerve injury, and pharmacological blockage or genetic knock out of P2X4R can dramatically reduce the pain hypersensitivity [16, 19]. Other evidences also showed that activating the functions of P2X4Rs of spinal microglia have important pathophysiological roles in evoking neuropathic pain [20]. The clone name of monoclonal antibody of complement receptor type 3 (OX42) is the antigen substance of spinal cord microglia, OX42 expression can be identified whether microglia is activated [21]. Electroacupuncture treatment has been reported to reduce spinal P2X4R expression in chronic constriction injury (CCI) rats [16]. But the regulation of acupuncture on P2X4 in DPN has not been reported.

In the present study, we used P2X4 and OX42 as targets to investigate the effects and underlying mechanisms of action of acupuncture, “filtering, clarification and excretion organs” in DPN. The results of this study evidently improve our understanding of the occurrence and the mechanisms that could prevent DPN.

## Methods

### Reagents and materials

#### Animals

Forty-eight adult SPF Sprague–Dawley male rats (180–220 g, 7 weeks) with fasting blood glucose < 7.0 mmo1/L were used and fed appropriately for 1 week. The rats were randomly divided into 2 groups: control group (n = 16), DPN model (n = 32). These were housed in groups of two to three per cage at a temperature of 22 ± 1 ℃ and 12 h light/dark cycle. Food and water were fed ad libitum. The study was approved by animal ethics committee [SCXK (ZHE) 2014-0001].

### Induction of DPN model

Based on the previous study [22] and making some modifications regarding the final composition of the diet, the rats in the model groups were fed a high calorie and high sugar diet composed of 72.5% normal diet along with 10.0% lard, 10.0% sucrose, 2.0% cholesterol, 0.5% sodium cholate, and 5.0% yolk powder. The tail vein blood was collected to measure fasting insulin (FINS) and fasting blood glucose (FBG). We used insulin sensitivity index (ISI, ISI = In (FINS × FBG)-1) as an important indicator for comparison before and after modeling [22–24], and we mainly referred to the index of random blood glucose in rats. Rats with the reduced ISI in the model group were given a single intraperitoneal injection of streptozotocin (STZ, 35 mg/kg, Sigma, USA). Rats in the control group received no STZ. Before injection, the STZ was dissolved in 1% of the citric acid buffer. The pH was controlled between 4.3–4.5. We could control the injection within 30 min and then proceed to the next manipulation. Rats in the model group with a FBG level of ≥ 11.1 mmol/L of the base value after 2 weeks were adopted as T2DM rat models [25, 26]. They were on high calorie and high sugar diet until the end of the experiments. After the diabetes model was established, mice weights, levels of fasting blood glucose, mechanical withdrawal threshold (MWT), thermal withdrawal latency (TWL), and biochemical indicators [triglyceride (TG), total cholesterol (TC), high-density lipoprotein-cholesterol (HDL-C) and low-density lipoprotein-cholesterol (LDL-C)] were measured for every 3 days. On the premise that blood glucose remains stable, and there is a statistical difference between mechanical pain and heat pain, it is considered to be a model of diabetic peripheral neuralgia (DPN). The neuralgia of rats appeared about 2 weeks after the model was manufactured.

### Acupuncture intervention

Eight rats were randomly selected from the DPN models to establish the DPN + Acu group. Acupuncture points, Feishu, Pishu and Shenshu (Additional file 1. Figure S1) were selected as per the acupoint map of the rats based on the literature [27]. These were administrated acupuncture for 20 min once daily for 14 days. During acupuncture, position of rat was fixed under restrain conditions so that the acupuncture can be administered smoothly.

### Mechanical withdrawal threshold (MWT) measurement

All rats were habituated to the testing environment for 3 days. Eight rats in each group were selected to measure mechanical withdrawal threshold according to the previous study [28]. The rats were placed in the plexiglass chamber for 30 min and von Frey fiber optic pain meter (Yuyan Instruments, Shanghai, China) was used to stimulate the hind paw of rats using the up-down method. Rats raising their legs, licking their feet or dodging were regarded as positive responses. The minimum grams of force required to get positive response was used as the pain threshold in each time. After modeling in rats, the corresponding data was tested every 3 days until the rats were killed. The MWT measurements were carried out five times in a row with intervals of 10 s each. All the behaviors were tested blindly.

### Thermal withdrawal latency (TWL) measurement

To evaluate thermal hyperalgesia, TWL was measured using RB-200 hot plate apparatus (Techman, Chengdu, Sichuang, China) with eight rats in each group as previous research [22]. At first, the hot plate was preheated to 55 °C. Then, the latency to the heat stimulus was measured for each rat by placing them on the hot plate. Time taken by the rat for hind limb licking or retracting was used as measurement. These were used as pain indicators. Response time was recorded for three times for each rat with an interval of more than 15 min. Data were recorded at the start of the experiment and at the end of 14^th^ day.

### Sample collection

After 14 days of acupuncture treatment as described above, sample collection was carried out. Prior to that, rats were made to fast for 12 h, but drinking water was available freely. 10 ml of abdominal aortic blood was aspirated using a syringe. This was injected into two test tubes containing an anticoagulant and two test tubes without it, centrifuged at 3000 rpm for 15 min. Plasma was in anticoagulated tubes and serum was in non-anticoagulated tubes. The supernatant was injected into the sterile Eppendorf (EP) tube and stored at -20 °C. Rats were deeply anesthetized with pentobarbital sodium (Sigma, St. Louis, MO, USA) by intraperitoneal injection. For spinal cord samples, eight rats were randomly selected from each group and were decapitated quickly on ice. Spinal cord extraction was divided into fresh spinal cord extraction and spinal cord extraction after perfusion (four rats per extraction). Both of our experiments were carried out. Fresh spinal cords were extracted as previous studies [29] and used for qRT-PCR and western blot assay. After perfusion, the spinal cord of each rat was extracted for the immunofluorescence detection. L4–6 spinal cords were collected and stored at − 80 °C.

### ELISA analysis

Serum levels of CXCR3, TNF-α, IL-1β, IL-6, glycosylated serum protein (GSP), TG, TC, HDL-C and LDL-C were measured at the beginning of the experiment and after 2 weeks using an enzyme linked immunosorbent assay (ELISA) kit (Abcam, USA), as per manufacturer’s directions.

### qRT-PCR assay

Total RNA samples from the spinal cords were obtained using TRIZOL extraction method (Invitrogen, California, USA). Sample concentrations were measured using NanoDrop ND-300 spectrophotometer (Aosheng, Hanzhou, China). 500 ng of total RNA from a sample was reverse-transcribed into cDNA by PrimeScript RT reagent Kit (TaKaRa, Dalian, China). These were then analyzed by qRT-PCR (CFX-Connect 96, Bio-Rad, Hercules, CA, USA) using SYBR FAST qPCR Master kit (KM4101, KAPA Biosystems, Wilmington, MA, USA). Primers for P2X4, OX42, β-actin were obtained from Tianyihuiyuan (Tianyihuiyuan, Wuhan, China) and its sequences were: *P2X4*-F: 5′-CTCATCCGCAGCCGTAAAGTGG-3′, *P2X4*-R: 5′-CACACGAACACCCACCCGATG-3′; *OX42*-F: 5′-GTGCTGGGAGATGTGAATGGAGAC-3′; *OX42*-R: 5′-GGTACTGATGCTGGCTACTGATGC-3′; β-actin-F: 5′-CGTTGACATCCGTAAAGAC-3′, β-actin-R: 5′-TAGGAGCCAGGGCAGTA-3′. The following thermocycling conditions were used: Initial denaturation at 95 °C for 3 min; 40 cycles of 95 °C for 5 s; annealing at 56 °C for 10 s; and a final extension at 72 °C for 25 s. Assays were carried out in triplicates, and the relative mRNA expression levels were quantified using the 2^−ΔΔCq^ method. β-actin was used as an internal control.

### Western blot analysis

Total protein samples extracted from the spinal cord (L4-6) were prepared by extraction using RIPA lysis buffer (Sigma, St. Louis, MO, USA). Protein concentrations were determined using the BCA Protein Assay Kit (0828A19, Leagene, Beijing, China). These proteins (50 μg each) were then run on 10% SDS-PAGE. Prestained protein marker (PageRulerTM Plus, 00752915, Thermo Scientific, Waltham, MA, USA) was included on each gel. Protein bands from the gel were transferred onto a polyvinylidene difluoride (PVDF) membrane. The PVDF membranes were activated with methanol prior to use. Nonspecific interactions with membrane were blocked using 5% non-fat milk in TBST (Sigma, St. Louis, MO, USA) for 2 h. The membranes were incubated with the corresponding primary antibodies (mouse anti-β-actin, mouse anti-P2X4, mouse anti-OX42, 1:1000, Cell Signaling Technology, MA, USA) overnight at 4 °C. After washing, the membranes were incubated in peroxidase-labeled secondary antibody (mouse anti-Rat IgG, 1:2000, 58,802, Cell Signaling Technology, MA, USA) for 2 h. The protein bands were visualized using ECL system (ATSAP2501, Abbkine, Redlands, CA, USA) and the intensities were analyzed by the gel imaging device (FCM, ProteinSimple, CA, USA).

### Immunofluorescence detection

The spinal cords (L4-6) of rats were dissected out. These were immediately postfixed in the same fixative for 3 h, and then consecutively immersed in 15% (0.15 g/ml) and 30% (0.3 g/ml) sucrose solutions for overnight at 4 °C. Tissues were embedded in optimal cutting medium, frozen, and then cut into 14 μm sections. Sections were mounted on glass slides, rinsed in Tris-buffered saline with Tween 20 (TBST; pH 7.4), blocked in 10% (0.1 g/ml) goat serum with 0.3% TritonX-100 for 1 h at 37 °C. These were then incubated with the primary antibodies, rat anti-mouse P2X4 (1:50; GTX54851, GeneTex, San Antonio, Texas, USA) or rat anti-mouse OX42 (1:50; GTX21211, GeneTex, San Antonio, Texas, USA) overnight at 4 °C. After 3 times of washing in PBS, these were incubated with Alexa Fluor 488-conjugated secondary antibody (mouse anti-rat IgG, 1:500, Abcam, Cambridge, UK) at 37 °C for 1 h. Staining was carried out using DAPI at 37 °C for 1 h. To quantitate the immunofluorescence, three sections from each specimen were analyzed. Signals were detected using an ECL system (ATSAP2501, Abbkine, Redlands, CA, USA).

### Statistical analysis

All statistical analyses were performed using GraphPad Prism 7.0 program (GraphPad, San Diego, CA, USA) and SPSS 22.0 Statistical Software (Chicago, IL, USA). Results are presented as the mean ± SEM (standard error of the mean). All experiments were carried out at least three times. The data of the two groups was assessed using the Student’s t-test, and the differences between the groups was analyzed by one-way analysis of variance (ANOVA), followed by Tukey’s multiple comparisons test. *P* < 0.05 was considered to be statistically significant.

## Results

### Acupuncture relieved the pain induced by DPN in rats

**Fig. 1:**
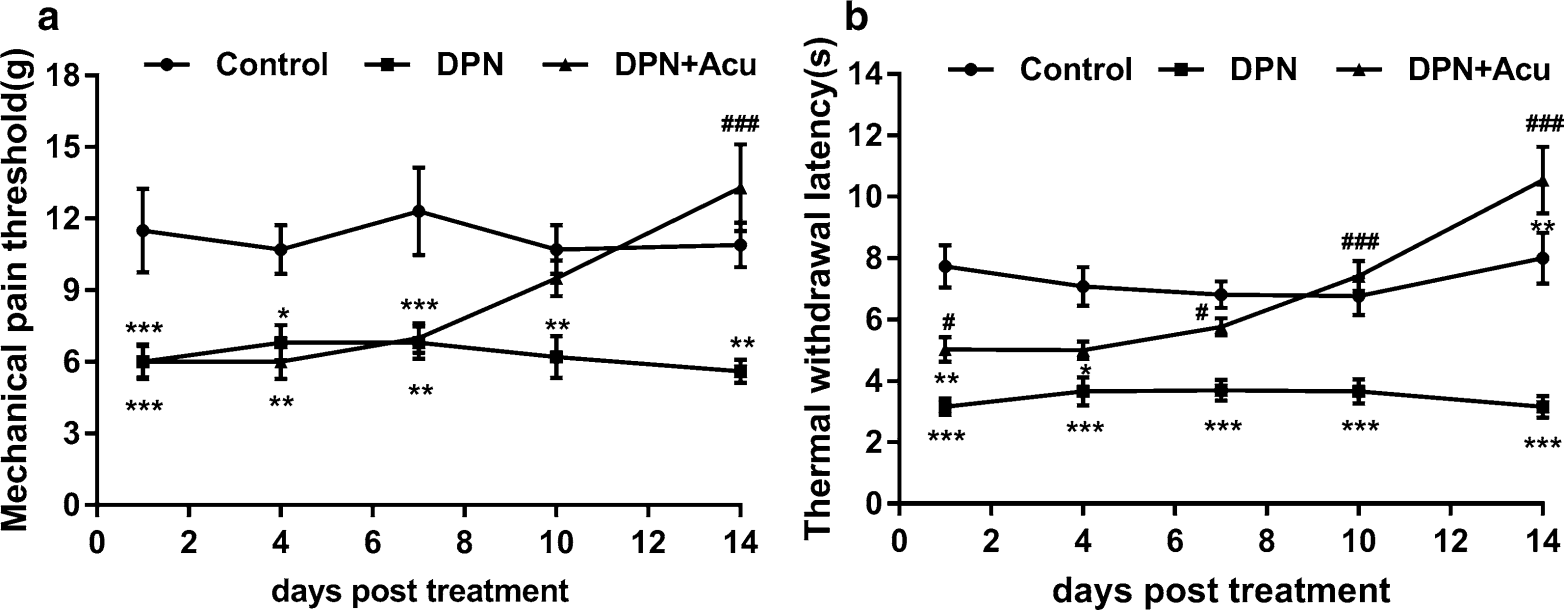
Acupuncture relieved the pain induced by DPN in rats. **a** Effect of acupuncture on the mechanical withdrawal threshold (MWT) of DPN rats. **b** Effect of acupuncture on the thermal withdrawal latency (TWL) of DPN rats. *P < 0.05, **P < 0.01, ***P < 0.001 vs. Control. ^##^*P* < 0.01, ^###^*P* < 0.001 vs. DPN. Acu, acupuncture

Post 14 days of acupuncture treatment at the “Feishu”, “Pishu” and “Shenshu” acupoints (as described in methods), we found that the acupuncture significantly (*P* < 0.05) decreased the MWT and TWL of DPN rats (Fig. 1a, b). This suggested for a significant analgesic effect of acupuncture in the DPN rats. However, acupuncture had no significant effect on body weights and FBG concentrations in DPN rats (*P* > 0.05; Additional file 2. Figure S2a, b).

### Acupuncture reduced the inflammation in DPN rats

**Fig. 2:**
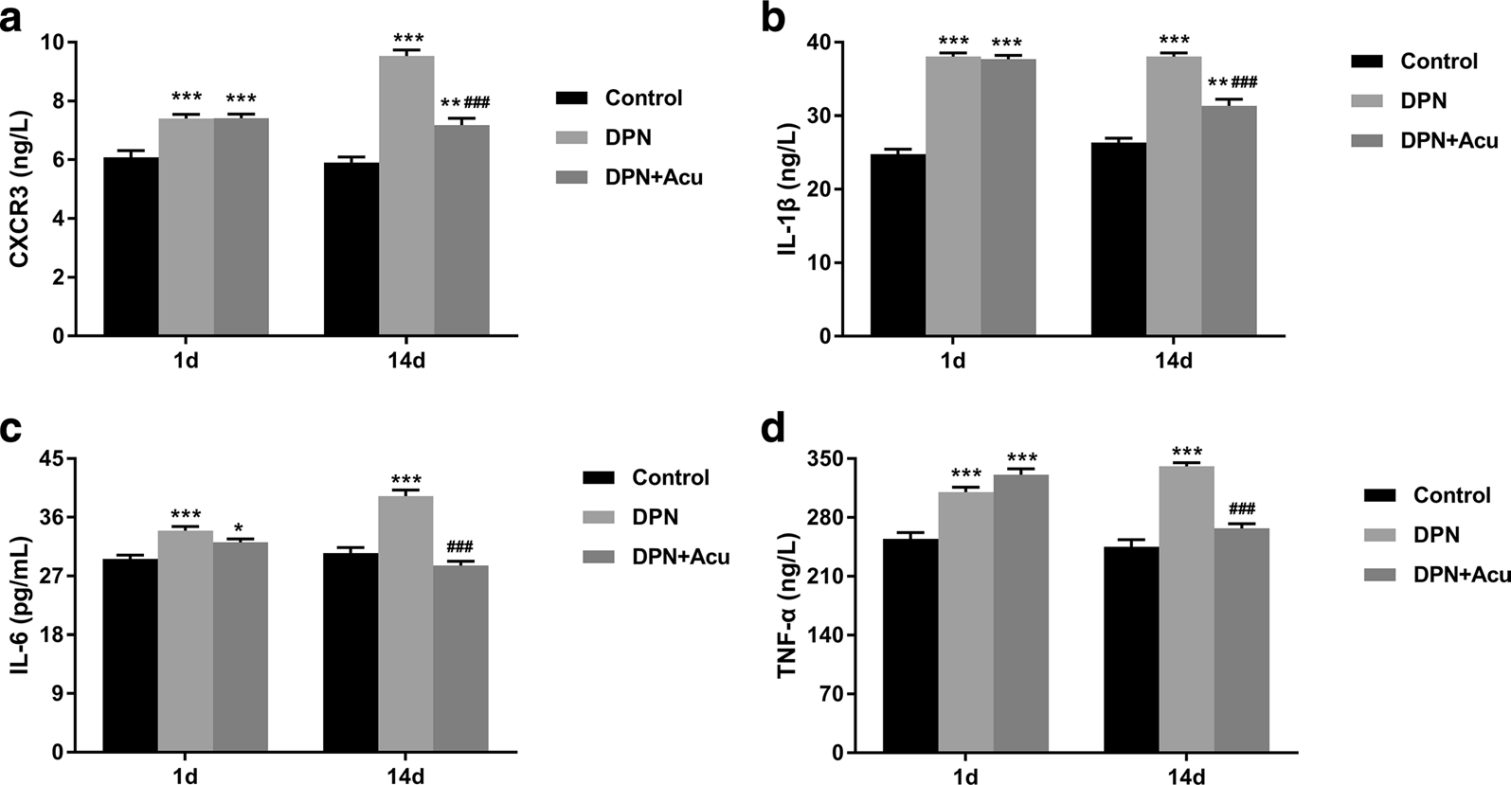
Acupuncture reduced the inflammation in DPN rats. **a** CXCR3, **b** IL-1β, **c** IL-6, and **d** TNF-α measured by ELISA on 1 and 14 days post-acupuncture. *P < 0.05, **P < 0.01, ***P < 0.001 vs. Control, ^###^*P* < 0.001 vs. DPN

As shown in Fig. 2, in comparison to the control group, levels of CXCR3, IL-1β, IL-6, and TNF-α were significantly increased in DPN group (*P* < 0.001). But, after providing 14 days of acupuncture treatment; the levels of these were decreased in DPN + Acu group compared to the DPN group (*P* < 0.001). These results revealed a significant effect of acupuncture treatment on reducing inflammation in DPN rats.

### Effects of acupuncture on GSP and lipid metabolisms in DPN rats

**Fig. 3:**
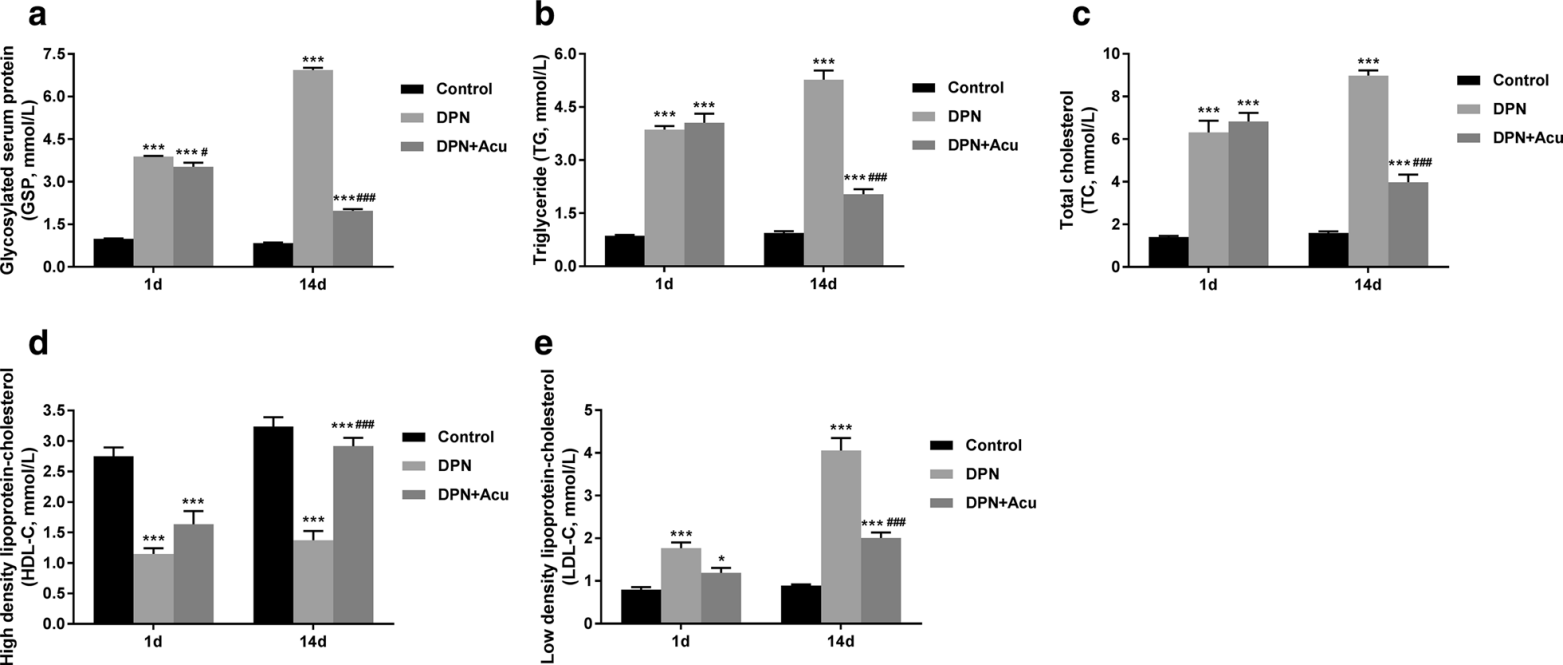
Effects of acupuncture on GSP and lipid metabolisms in DPN rats. **a** Glycosylated serum protein (GSP), **b** triglyceride (TG), **c** Total cholesterol (TC), **d** high-density lipoprotein-cholesterol (HDL-C) and **e** Low-density lipoprotein-cholesterol (LDL-C) measured by ELISA at the beginning (1 d) and at the end of acupuncture treatment (14 d). *P < 0.05, ***P < 0.001 vs. Control; ^#^*P* < 0.05, ^###^*P* < 0.001 vs. DPN

The results of ELISA showed that the glycosylated serum protein (GSP), triglyceride (TG), total cholesterol (TC), and low-density lipoprotein-cholesterol (LDL-C) levels in DPN group were significantly increased compared to the control group (*P* < 0.001) (**F**ig. 3a, b, c, e). However, as shown in Fig. 3d, high-density lipoprotein-cholesterol (HDL-C) level in DPN group was significantly increased compared with cControl group (*P* < 0.001). Post 14 days treatment, acupuncture could significantly decrease the levels of GSP, TG, TC, and LDL-C but increased the HDL-C in DPN rats (*P* < 0.001). These results suggested that acupuncture treatment could regulate the lipid metabolism, having an important effect on the inflammation in DPN rats (Fig. 3).

### Acupuncture reduced P2X4 and OX42 expression in DPN rats

**Fig. 4:**
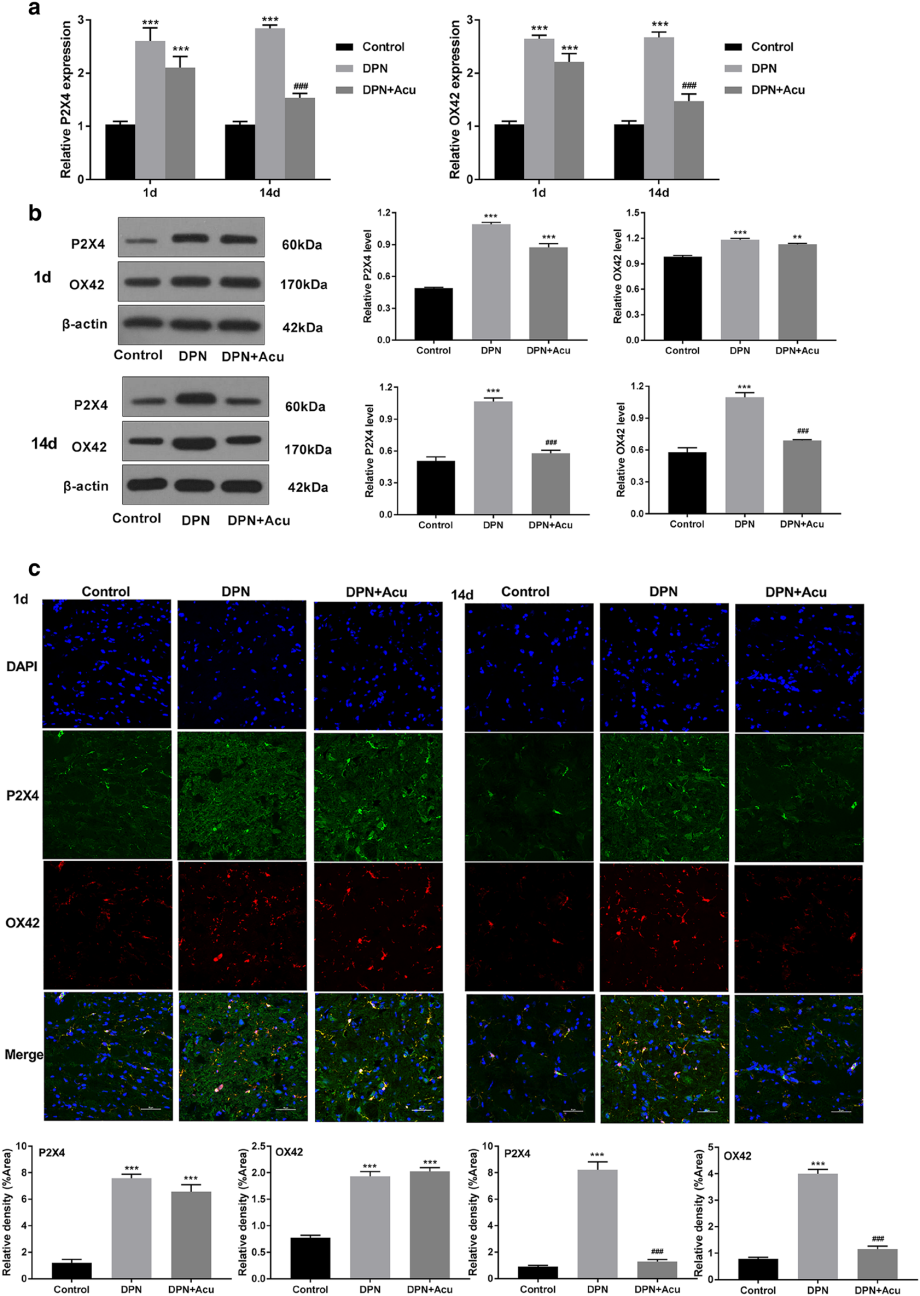
Acupuncture reduced P2X4 and ox42 expression in DPN rats. **a** qRT-PCR showing relative mRNA level of P2X4 and OX42 on different days post-acupuncture. **b** Representative western blot of P2X4 and OX42 protein levels on 1 and 14 days post-acupuncture. **c** Representative photomicrographs of immunofluorescence of P2X4 (green) and OX42 (red) at the beginning (1 d) and at the end of acupuncture treatment (14 d) (Original magnification × 400) and its fluorescence intensity. *P < 0.05, **P < 0.01, ***P < 0.001 vs. Control, ^#^*P* < 0.05, ^###^*P* < 0.001 vs. DPN

When compared to the control group at day 1 and after 14 days of treatment, significant changes were observed between DPN and DPN + Acu groups in both markers (P2X4 and OX42). qRT-PCR assay showed that the expression level of P2X4 and OX42 in DPN group was increased compared with that in the control group (*P* < 0.001). Between the DPN and DPN + Acu group, there were no significant changes at day 1. However, acupuncture treatment could reduce the P2X4 and OX42 expression of DPN rats (*P* < 0.001, Fig. 4a). We also found similar results in western blot analysis (Fig. 4b). Furthermore, the results from immunofluorescence assay also indicated for significantly suppressed number of P2X4R + in microglia (identified by P2X4 and OX42) upon acupuncture treatment. Figure 4c reveals that in contrast to the control group, the spinal cord OX42 (red) and P2X4 (green) staining in the DNP group was significantly enhanced, the number of microglia cells was increased. But, after acupuncture treatment, in the DPN + Acu group, both OX42 and P2X4 staining were decreased. The numbers of marked microglia cells were also reduced compared with DNP group (Fig. 4c). These results suggested that acupuncture leads to the reduction in P2X4 expression levels in DPN rat.

## Discussion

**Fig. 5:**
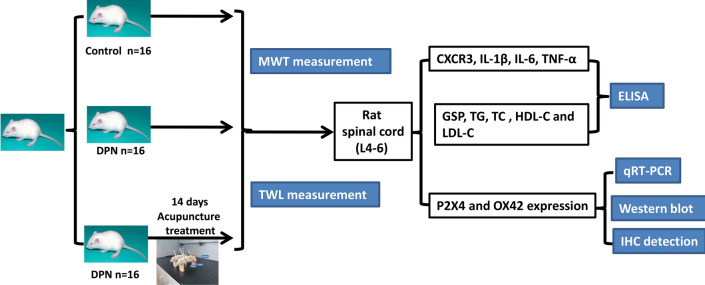
The research route of this study

This study was designed to investigate the effects of acupuncture treatment in DPN using T2DM rat models (Fig. 5). We found that the acupuncture treatment improves DPN symptoms via inhibiting P2X4 expression in spinal microglia. This is also marked by decreased mechanical allodynia.

Previous studies showed that a long-term high-fat, high-sugar diet can be used to induce obesity in rodents that ultimately establishes a model of type II diabetes through the elevation of blood glucose levels [22]. Although it is a long-lasting animal model, the methodology to be followed can be optimized and improved [30, 31]. To circumvent this, an optimal, stable, and practical animal model was established. Here, an optimal rat model of type II diabetes is obtained by using a single adjusted dose of STZ, along with improved high-sugar and high-fat diet formulations [22, 32]. STZ simulates insulin resistance by islet destruction that mirrors the typical pathogenesis seen in type II diabetes [33]. Furthermore, this method only requires approximately 2 months to obtain an insulin resistance model, thus greatly reduces the time and cost in contrast to a transgenic or genetic model [22]. Once type II diabetes has been established and has progressed, the pain threshold in these diabetic rats is significantly reduced within 2 weeks [22]. Thereby confirms establishing a model of DPN that exhibits neuropathic pain-related indicators.

In order to validate the model of type II diabetes that exhibits neuropathic pain [34], we performed additional studies to find its stabilization time. Pain threshold and the related indicators were observed in the rats of a subgroup B2 at 4 weeks (2 weeks after the model was established) after STZ injection. The rats in this group were also treated with STZ at the optimal dose (35 mg/kg). The results showed that, compared with the control group, the pain threshold in DPN rats was also significantly decreased. This demonstrates the accuracy and stability of the novel rat model of type II diabetic neuropathic pain that we developed in this study.

DPN is a recognized microvascular complication of diabetes, and its main etiology is loss of nourishment in tendons, vessels, and skin caused by blood stasis [2]. Based on the pathogenesis of DPN, we formulated the treatment plan of “adjusting internal organs and dredging channel” acupuncture. Feishu (BL13), Pishu (BL20), and Shenshu (BL23) are back-shu points of the lung, spleen, and kidney that can regulate the function of these organs, and achieve the purpose of strengthening the spleen and benefiting the lung, enriching the kidney and strengthening Yang-qi [35, 36].

Acupuncture that originated in China is now an accepted alternative therapy for various diseases in both Eastern and Western countries. Acupuncture-induced analgesia has been widely used to alleviate various types of pains, particularly the chronic pain [12, 37]. Acupuncture can also significantly alleviate myelinated nerve fiber damage, further indicating the efficacy of acupuncture treatment of DPN in this research, which is in agreement with the previous reports [38, 39]. Studies show that DPN models have higher blood lipid levels (GSP, TG, TC, and LDL-C) [30–32]. In this study, after acupuncture treatment, we found that the levels of these were decreased; furthermore, acupuncture also improved MWT and TWL response in DPN rats. Based on these data, we speculated that acupuncture (Feishu, Pishu, and Shenshu) could relieve the DPN. The clinical efficacy of acupuncture in treating DPN is greatly established, but the underlying mechanism of action, the role of P2X4 on MWT, TWL in DPN rats would need further research.

A large number of studies have shown that spinal microglia play a key role in the occurrence and development of DPN [40]. Microglia are also the main sources of inflammatory factors TNF-α, IL-1β, and IL-6 [41]. Upon stimulation of peripheral nerve injury, spinal microglia is activated that leads to the secretion of inflammatory factors. This could be mediated via the p38 mitogen-activated protein kinase (p38MAPK) signaling pathway; moreover, inflammatory response can also activate p38MAPK to promote the activation of spinal microglia [42]. This generates a positive feedback pathway between inflammatory factors and microglia [43]. The inflammatory factors such as TNF-α, IL-1β, and IL-6 can also affect sodium and calcium channels on the cell membrane [44, 45]. These rapidly increase the excitability of neurons and further leads to the increase in the channels conductivity [40]. Simultaneously, inflammatory factors can also destroy glycosylated myelin, that triggers demyelination of nerves, causing central sensitization and pain [41]. In this study, after 14 days of acupuncture treatment, we found that compared with the DNP group, pain was relieved and expression of inflammatory factor was reduced in the DNP + Acu group. This effect could also be due to the altered release of the inflammatory factors.

A previous report showed that P2X4 receptors of activated microglia in the spinal cord are involved in the pathogenesis of neuropathic pain [46], and reported that P2X4 receptors expression was increased by spinal microglia during neuropathic pain [47]. P2 receptors activate microglia through ATP receptors to initiate cellular response. As we know, OX42 is a cell surface marker of microglia activation [48]. When peripheral nerves were injured, microglia can be changed, including morphological changes and increased expression of microglia markers OX42 [49]. Another research has showed that peiminine treatment can down-regulate the expression of OX-42 in the LPS-induced PD rat model by inhibiting microglial activation [50]. In our research too, we found that P2X4 and OX42 were over expressed in DPN group, but their expression was significantly suppressed after acupuncture treatment. Therefore, these results suggest that acupuncture could relieve DPN via regulating P2X4 in spinal microglia.

## Conclusions

In summary, our study showed that DPN results in up-regulation of P2X4 expression in DPN rats. Acupuncture could relieve DPN in our model. These effects could be result of reduced inflammation, GSP and lipid metabolisms. Furthermore, it revealed that acupuncture can also down-regulate the expressions of P2X4 and OX42, thereby alleviating DPN. Overall, these findings advocate for acupuncture treatment in the management of DPN.

### Supplementary information

**Additional file 1: Figure S1.** Representative image of manipulation with acupuncture at the 3 chosen points (Feishu, Pishu and Shenshu) in rat.

**Additional file 2: Figure S2.** Effect of acupuncture on the body weights (**a**) and fasting blood glucose (**b**) of DPN rats. ***P < 0.001 vs. Control.

## Data Availability

All data generated or analyzed during this study are included in this published article.

## Abbreviations

ANOVA: One-way analysis of variance
DM: Diabetes mellitus;
DPN: Diabetic peripheral neuropathy
FBG: Fasting blood glucose (FBG)
FINS: Fasting insulin
GSP: Glycosylated serum protein
HDL-C: High-density lipoprotein-cholesterol
ISI: Insulin sensitivity index
LDL-C: Low-density lipoprotein-cholesterol
MWT: Mechanical withdrawal threshold
STZ: Streptozotocin
TC: Total cholesterol
TCM: Traditional Chinese medicine
TG: Triglyceride
TWL: Thermal withdrawal latency
