# Supplementary figures and images for: Acupuncture attenuates the development of diabetic peripheral neuralgia by regulating P2X4 expression and inflammation in rat spinal microglia
Source: J Physiol Sci. 2020 Sep 23;70:45. doi: 10.1186/s12576-020-00769-8 (PMC10717860; doi:10.1186/s12576-020-00769-8)

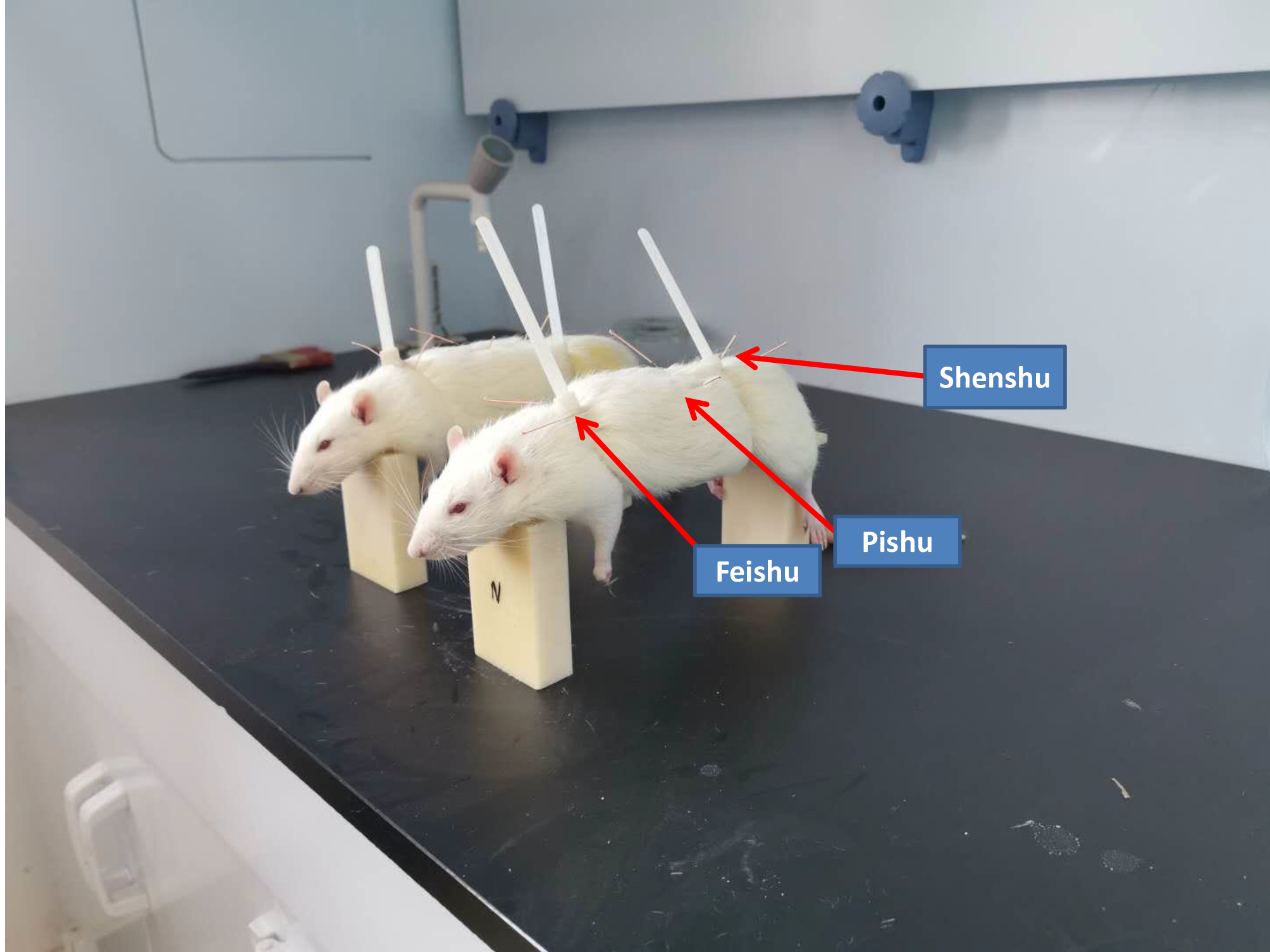

Supplement: Supplementary file 1 — Additional file 1: Figure S1. Representative image of manipulation with acupuncture at the 3 chosen points (Feishu, Pishu and Shenshu) in rat. [file 12576_2020_769_MOESM1_ESM.tif]

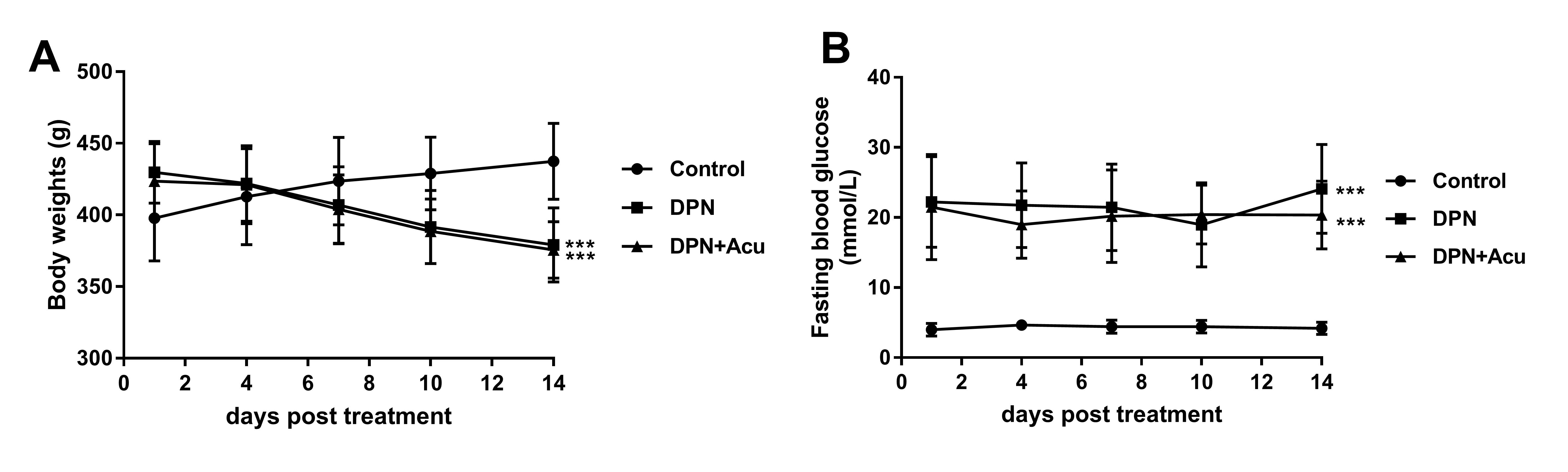

Supplement: Supplementary file 2 — Additional file 2: Figure S2. Effect of acupuncture on the body weights (a) and fasting blood glucose (b) of DPN rats. ***P < 0.001 vs. Control. [file 12576_2020_769_MOESM2_ESM.tif]
